# The Relationship Between Mitral Annular Calcification and Controlling Nutritional Status Score

**DOI:** 10.21470/1678-9741-2020-0443

**Published:** 2022

**Authors:** İpek Büber, Mehmet Koray Adalı, Derya Kaya, İsmail Doğu Kılıç, Samet Yılmaz, Dursun Dursunoğlu

**Affiliations:** 1 Department of Cardiology, Faculty of Medicine, Pamukkale University, Denizli, Turkey.

**Keywords:** Mitral Valve, Heart Valve Diseases, Chronic Diseases, Nutrition Assessment, Nutrition Status, Severity of Illness Index

## Abstract

**Introduction:**

The relationship between mitral annular calcification (MAC) and the controlling nutritional status (CONUT) score has not been previously studied. In this study, we investigated the relationship between MAC and CONUT score to evaluate the nutritional status of patients with MAC.

**Methods:**

A total of 275 patients, including 150 patients with MAC and 125 patients without MAC, who presented to a cardiology outpatient clinic were enrolled in the study.

**Results:**

There was no difference in the CONUT score between the two groups. Correlation analysis indicated that CONUT score was positively correlated with left atrial (LA) diameter (r=0.190, *P*=0.020) and interventricular septum thickness (r=0.179, *P*=0.028) in the MAC+ group. In multivariate regression analysis, only LA diameter (odds ratio 95% confidence interval = 1,054-1,189, *P*=0.0001) was independently associated with MAC.

**Conclusion:**

The present study investigated the relationship between CONUT score and MAC for the first time in the literature. We demonstrated that CONUT score was not significantly higher in patients with MAC without chronic diseases. However, CONUT score was correlated with LA diameter in patients with MAC. We therefore conclude that, for patients admitted with MAC and high LA diameter, CONUT is a valuable nutritional and inflammatory status index.

**Table:** 

Abbreviations, acronyms & symbols	
CI	= Confidence interval
CONUT	= Controlling nutritional status
EF	= Ejection fraction
HDL	= High-density lipoprotein
IVS	= Interventricular septum
LA	= Left atrial
LDL	= Low-density lipoprotein
LVED	= Left ventricular end-diastolic
LVES	= Left ventricular end-systolic
MAC	= Mitral annular calcification
MPV	= Mean platelet volume
MR	= Mitral regurgitation
NLR	= Neutrophil/lymphocyte ratio
RDW	= Red cell distribution width
SD	= Standard deviation

## INTRODUCTION

Mitral annular calcification (MAC) is a degenerative process caused by lipid and calcium storage in the mitral valve apparatus, which can affect mitral valve functions^[1]^. MAC is associated with atherosclerotic processes in different regions such as carotid artery disease, coronary artery disease, and aortic atheroma^[2]^. Several studies have shown a relationship between MAC and mean platelet volume (MPV)^[3]^, red cell distribution width (RDW)^[4]^, platelet/lymphocyte ratio^[5]^, monocyte/high-density lipoprotein (HDL) ratio^[6]^, and neutrophil/lymphocyte^[7]^ ratio (NLR), all of which may be related to cardiovascular risk factors. The nutritional status of patients with MAC has not been adequately studied to date. However, Tanik and Pamukcu showed that the prognostic nutritional index was lower in patients with MAC^[8]^. The controlling nutritional status (CONUT) score is a simple and useful tool to identify patients at risk of developing nutrition-related complications^[9]^. The CONUT score uses two biochemical parameters (serum albumin and cholesterol levels) and one immune parameter (total lymphocyte count) to assess nutritional status and inflammation. Soft-tissue calcification like MAC occurs with chronic inflammation. The CONUT score has been shown to predict short-term and long-term prognoses in patients with heart failure^[10]^. In this study, we investigated the relationship between MAC and the CONUT score to evaluate the nutritional status of patients with MAC.

## METHODS

### Patient Selection

The study was performed in compliance with the principles outlined in the Declaration of Helsinki and was approved by the Pamukkale University’s local ethics committee (approval number 29683). All patients who were admitted to the Pamukkale University’s Department of Cardiology outpatient clinic between January and December 2019 were evaluated retrospectively. The study included 150 patients with MAC (MAC+) and a control group of 125 patients without MAC (MAC-). The composition of groups was similar in terms of age and sex. Exclusion criteria were diabetes mellitus, hypertension, chronic renal or liver disease, moderate to severe mitral stenosis, aortic stenosis and aortic regurgitation, malignancy, history of systemic or pulmonary embolism, chronic hematological diseases, acute or chronic inflammatory disease, autoimmune disease, current use of anticoagulants, presence of a prosthetic valve, permanent and paroxysmal atrial fibrillation, congestive heart failure, or history of immunosuppressant usage. Glucose, creatinine, all lipid parameters, and complete blood count values were obtained from hospital records.

### Calculation of the CONUT Score

In this study, the CONUT score was used to evaluate the nutritional status of patients with MAC. This score uses three parameters: the serum albumin level (g/dL), total cholesterol level (mg/dL), and total lymphocyte count (count/ml). Thus, the CONUT score provides an evaluation of protein reserves, calorie depletion, and immune defense. Score values were assigned to different ranges of laboratory measurements as follows: serum albumin ≥ 3.5 was zero point, 3-3.49 was two points, 2.5-2.99 was four points, and < 2.5 was six points; lymphocytes ≥ 1600 was zero point, 1200-1599 was one point, 800-1199 was two points, and < 800 was three points; total cholesterol ≥ 180 was zero point, 140-179 was one point, 100-139 was two points, and < 100 was three points. A score of 0-1 was defined as normal, 2-4 was defined as mild CONUT, 5-8 was defined as medium CONUT, and ≥ 9 was severe CONUT. A higher CONUT score indicates a worse nutritional status^[9]^.

### Statistical Analysis

The IBM Corp. Released 2015, IBM SPSS Statistics for Windows, version 23.0, Armonk, NY: IBM Group software was used for statistical analysis. Continuous variables are shown as mean ± standard deviation and categorical variables are given as number and percentage. Kolmogorov-Smirnov test was used to examine the normal distribution of data. Student’s *t*-test or Mann-Whitney U test was used for numerical variables and Chi-square test was used for analysis of categorical variables. The relationships between normally and non-normally distributed continuous variables were analyzed by using Pearson’s or Spearman’s correlation analysis. The independent predictors for the presence of MAC were analyzed by using logistic regression analysis. Possible confounding factors were tested with a univariable regression analysis, and those with *P*<0.1 were tested with a multivariable logistic regression analysis. A two-sided *P*-value of < 0.05 was considered statistically significant.

## RESULTS

Demographic characteristics, biochemical parameters, and whole blood parameters of the study groups are summarized in Table 1, and echocardiographic parameters and CONUT scores are summarized in Table 2. There were no differences in age, sex, glucose values, and creatinine values between the two groups (Table 1). Total cholesterol (MAC- 176.39±45.12; MAC+ 187.53 ± 46.03, *P*=0.022), HDL (MAC­- 43.35±11.59; MAC+ 48.20 ± 13.03, *P*=0.003), and NLR were significantly higher in the MAC+ group; lymphocyte count was significantly higher MAC- group (MAC- 2.4±0.9; MAC+ 2.2 ±1.1, *P*=0.022). In the MAC+ group, mild mitral regurgitation (MR) was observed in 85.9% (n = 128), moderate MR was observed in 10.1% (n = 15), and severe MR was observed in 3% (n = 2) of the patients. In the MAC- group, mild MR was observed in 92.8% (n = 116) and moderate MR was observed in 2.4% (n = 3) of the patients; there was no severe MR in the MAC- group.

**Table 1:** Demographic characteristics, biochemical parameters, and whole blood parameters of the groups.

Parameters	MAC- (n=125) Mean ± SD (min - max)	MAC+ (n=150) Mean ± SD (min - max)	*P*-value
Age, years	70.6 ± 8.3 (55 - 91)	72.1 ± 10.2 (33 - 97)	0.075^β^
Male patients (n/%)	48/38.4	47/31.3	0.22^δ^
Glucose (mg/dl)	113 ± 45 (67 - 158)	114 ± 23 (90 - 138)	0.122^β^
Creatinine (mg/dl)	1.1 ± 0.7 (0.49 - 1.29)	1.1 ± 0.8 (0.45 - 1.3)	0.572^β^
Total cholesterol (mg/dl)	176.4 ± 45.1 (86 - 333)	187.5 ± 46 (40 - 319)	0.022*^β^
LDL-cholesterol (mg/dl)	114 ± 28.1 (48 - 231)	117.9 ± 30 (29 - 230)	0.208^β^
HDL-cholesterol (mg/dl)	43.3 ± 11.6 (20 - 74)	48.2 ±13 (21 - 123)	0.003*^β^
Triglyceride (mg/dl)	167 ± 86.8 (46 - 676)	158.9 ± 73.9 (52 - 459)	0.546^β^
Total leukocyte (K/uL)	8.4 ± 2.4 (1.87 - 15.1)	8.6 ± 3.2 (4.1 - 28.8)	0.649^β^
Neutrophil (K/uL)	5.3 ± 2.1 (1.87 - 15.1)	5.6 ± 2.8 (1.68 - 26)	0.558^β^
Lymphocyte (K/uL)	2.4 ± 0.9 (0.75 - 8.37)	2.2 ± 1.1 (0.1 - 10.9)	0.022*^β^
Monocyte (K/uL)	0.5 ± 0.2 (0.1 - 1.25)	0.5 ± 0.2 (0.08 - 1.32)	0.447^β^
NLR	2.6 ± 2 (0.42 - 17.98)	3 ± 2.3 (0.58 - 19.11)	0.045*^β^
Platelet/lymphocyte ratio	132.1 ± 84.7 (35.4 - 806.6)	140 ± 79.8 (8.3 - 646.1)	0.456^β^
Hemoglobin (g/dl)	12.7 ± 1.8 (7,9 - 17,1)	12.3 ± 2.1 (6.6 - 16.5)	0.095^α^
Monocyte /HDL ratio	0.01 ± 0.006 (0,00 - 0,04)	0.012 ± 0.005 (0,00 - 0,03)	0.363^β^
Platelets (K/uL)	274.5 ± 88.0 (73 - 605)	255.9 ± 80 (13,3 - 539)	0.077^β^
RDW (%)	14.4 ± 1.8 (11.8 - 25.9)	15.6 ± 17.1 (11.5 - 223)	0.611^β^
MPV (fL)	9.4 ± 1.2 (7.5 - 15.4)	9.6 ± 1.1 (7.4 - 15.3)	0.113^β^

HDL=high-density lipoprotein; LDL=low-density lipoprotein; MAC=mitral annular calcification; MPV=mean platelet volume; NLR=neutrophil/lymphocyte ratio; RDW=red blood cell distribution width; SD=standard deviation

* *P*<0.05

α Independent samples *t*-test

β Mann-Whitney U test

δ Chi-square test

**Table 2:** Echocardiography findings and CONUT score.

Parameters	MAC- Mean ± SD	MAC+ Mean ± SD	*P*-value
LA diameter (mm)	36 (36.71 ± 4,03)	39 (39.32 ± 4,77)	0.0001*^β^
EF (%)	62 (58.64 ± 3,38)	60 (58.45 ± 3.11)	0.657^β^
LVED diameter (mm)	47 (47.38 ± 4,36)	47 (47.24 ± 4.62)	0.928^β^
LVES diameter (mm)	30 (31.78 ± 5.33)	30 (31.22 ± 4.63)	0.657^β^
IVS (mm)	11 (10.9 ± 1.64)	11 (11.26 ± 2.11)	0.201^β^
Posterior wall thickness	10 (10.37 ± 1.22)	10 (10.72 ± 1.43)	0.042*^β^
CONUT score	1 (1.24 ± 1.39)	1 (1.43 ± 1.7)	0.609^β^
**CONUT score classification**			
0-1	82 (65.6%)	100 (66.7%)	0.927^δ^
02/abr	38 (30.4%)	43 (28.7%)	
≥ 5	5 (4%)	7 (4.7%)	

CONUT=controlling nutritional status; EF=ejection fraction; IVS=interventricular septum; LA=left atrial; LVED=left ventricular end-diastolic; LVES=left ventricular end-systolic; MAC=mitral annular calcification; SD=standard deviation

* *P*<0.05

β Mann-Whitney U test

δ Chi-square test

Left atrial (LA) diameter (MAC- 36.71±4.03; MAC+ 39.32 ± 4.77, *P*=0.0001) and posterior wall thickness (MAC- 10.37 ± 1.22; MAC+ 10.72 ± 1.43, *P*=0.042) were significantly higher in the MAC+ group. There was no difference in the CONUT score between the two groups (Table 2). In the MAC+ group, the CONUT score was 0-1 in 66.7% (n=100), 2-4 in 28.7% (n=43), and ≥ 5 in 4.7% (n=7) of the patients. In the MAC- group, the CONUT score was 0-1 in 65.6% (n=82), 2-4 in 30.4% (n=38), and ≥ 5 in 4% (n=5) of the patients.

**Fig. 1:**
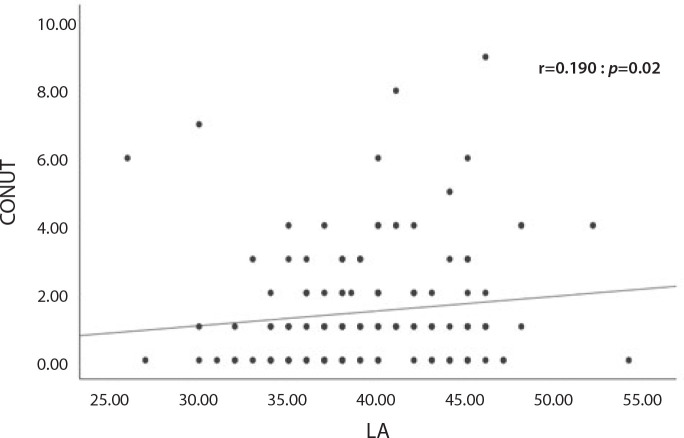
Correlation analysis between left atrial (LA) diameter and controlling nutritional status (CONUT) score in MAC+ groups. MAC=mitral annular calcification

Correlation analysis indicated that the CONUT score was positively correlated with LA diameter (r=0.190, *P*=0.020) and interventricular septum thickness (r=0.179, *P*=0.028) in the MAC+ group (Figure 1; Table 3). Multivariate regression analysis showed that only LA diameter (odds ratio 95% confidence interval = 1.054-1.189, *P*=0.0001) was independently associated with MAC (Table 4).

**Table 3:** Parameters correlated with CONUT score in MAC- and MAC+ patients.

	MAC- r/*P*-value	MAC+ r/*P*-value
LA diameter	0.052/0.568	0.190*/0.020*
IVS thickness	0.018/0.842	0.179*/0.028

CONUT=controlling nutritional status; IVS=interventricular septum; LA=left atrial; MAC=mitral annular calcification

* *P*<0.05

r=Spearman’s correlation coefficient

**Table 4:** Univariate and multivariate logistic regression analysis of parameters predicting the existence of MAC.

Variables	Univariate		Multivariate	
	Odds ratio (95% CI)	*P*-value	Odds ratio (95% CI)	*P*-value
Mitral regurgitation	3.09 (1.354 - 7,053)	0.007*	2.29 (0.966 - 5.420)	0.06
CONUT score	1.08 (0.928 - 1,266)	0.309		
LA diameter	1.14 (1.078 - 1,211)	0.0001*	1.12 (1.054 - 1.189)	0.0001*
Ejection fraction	0.98 (0.913 - 1.057)	0.633		
IVS thickness	1.11 (0.970 - 1.277)	0.127		
Posterior wall thickness	1.23 (1.019 - 1.487)	0.031*	1.19 (0.978 - 1.460)	0.082
LVED diameter	0.993 (0.942 - 1.047)	0.791		
LVES diameter	0.977 (0.931 - 1.026)	0.357		

CI=confidence interval; CONUT=controlling nutritional status; IVS=interventricular septum; LA=left atrial; LVED=left ventricular end-diastolic; LVES=left ventricular end-systolic; MAC=mitral annular calcification

## DISCUSSION

In the present study, we used the CONUT score to investigate the nutritional status in patients with MAC. CONUT score was not statistically higher in the MAC+ group compared to the MAC- group. However, a significant positive correlation was found between CONUT score and LA diameter, which is associated with chronic conditions and poor outcomes in cardiovascular disease, in the MAC+ patients^[11]^. In other words, LA diameter was independently associated with poor nutritional status in MAC+ patients, even without chronic disease.

In previous studies, MAC was associated not only with coronary atherosclerosis, but also with aortic atheroma and carotid artery disease^[2,12]^. MAC is considered a form of atherosclerosis due to risk factors and pathology similar to atherosclerotic lesions. Several studies have found a relationship between MAC and inflammatory parameters such as C-reactive protein, intracellular adhesion molecule-1, and interleukin-6^[13,14]^. A significant relationship has also been shown between MAC and NLR, which is an indirect marker of inflammation^[7]^. Consistent with prior studies, we found that NLR was significantly higher in patients with MAC compared with patients without MAC. However, although previous studies demonstrated a relationship between MAC and MPV^[3]^, RDW^[4]^, monocyte/HDL ratio^[6]^, platelet/lymphocyte ratio^[5]^, and prognostic nutritional index^[8]^, we did not observe a similar relationship between MAC and these parameters.

LA diameter has been determined as a prognostic marker for adverse cardiovascular events^[11,15-19]^. In addition, high CONUT score is associated with systemic inflammation and poor outcomes in patients with chronic heart failure^[20]^. In our study, in patients with MAC, CONUT score was positively correlated with LA diameter; in patients without MAC, there was no relationship between CONUT score and LA diameter. We suggest that inflammation may contribute to increased LA diameter in MAC+ patients.

### Limitations

The current study has certain limitations. First, it does not provide prognostic data due to its cross-sectional design. Second, it was a single-center study that included a relatively small number of patients. Third, a number of relevant parameters, including LA volume index and body mass index, were not available for all patients. Therefore, other nutritional indices such as the geriatric nutritional index could not be evaluated.

## CONCLUSION

In conclusion, the present study investigated the relationship between CONUT score and MAC for the first time in the literature. We demonstrated that CONUT score was not significantly higher in patients with MAC when compared with control patients without MAC. However, the CONUT score was correlated with LA diameter in patients with MAC. In MAC+ patients, greater LA diameter was associated with poor nutritional process and inflammation. We therefore conclude that, for patients admitted with MAC and increased LA diameter, CONUT is a valuable nutritional status index. Prospective studies with larger patient populations could confirm the relationship between MAC and CONUT score and evaluate prognostic implications.
